# Regionally Distinct Responses of Microglia and Glial Progenitor Cells to Whole Brain Irradiation in Adult and Aging Rats

**DOI:** 10.1371/journal.pone.0052728

**Published:** 2012-12-26

**Authors:** Kun Hua, Matthew K. Schindler, Joseph A. McQuail, M. Elizabeth Forbes, David R. Riddle

**Affiliations:** 1 Department of Neurobiology and Anatomy, Wake Forest School of Medicine, Winston-Salem, North Carolina, United States of America; 2 Program in Neuroscience, Wake Forest School of Medicine, Winston-Salem, North Carolina, United States of America; 3 Department of Neurology, University of Pennsylvania Health System, Philadelphia, Pennsylvania, United States of America; St. Jude Children's Research Hospital, United States of America

## Abstract

Radiation therapy has proven efficacy for treating brain tumors and metastases. Higher doses and larger treatment fields increase the probability of eliminating neoplasms and preventing reoccurrence, but dose and field are limited by damage to normal tissues. Normal tissue injury is greatest during development and in populations of proliferating cells but also occurs in adults and older individuals and in non-proliferative cell populations. To better understand radiation-induced normal tissue injury and how it may be affected by aging, we exposed young adult, middle-aged, and old rats to 10 Gy of whole brain irradiation and assessed in gray- and white matter the responses of microglia, the primary cellular mediators of radiation-induced neuroinflammation, and oligodendrocyte precursor cells, the largest population of proliferating cells in the adult brain. We found that aging and/or irradiation caused only a few microglia to transition to the classically “activated” phenotype, e.g., enlarged cell body, few processes, and markers of phagocytosis, that is seen following more damaging neural insults. Microglial changes in response to aging and irradiation were relatively modest and three markers of reactivity - morphology, proliferation, and expression of the lysosomal marker CD68- were regulated largely independently within individual cells. Proliferation of oligodendrocyte precursors did not appear to be altered during normal aging but increased following irradiation. The impacts of irradiation and aging on both microglia and oligodendrocyte precursors were heterogeneous between white- and gray matter and among regions of gray matter, indicating that there are regional regulators of the neural response to brain irradiation. By several measures, the CA3 region of the hippocampus appeared to be differentially sensitive to effects of aging and irradiation. The changes assessed here likely contribute to injury following inflammatory challenges like brain irradiation and represent important end-points for analysis in studies of therapeutic strategies to protect patients from neural dysfunction.

## Introduction

Approximately 200,000 patients in the United States are treated each year with partial- or whole-brain irradiation (WBI) [1]–[3], and half or more that survive ≥six months develop neural dysfunction due to radiation-induced injury of normal brain tissue [4], [5]. Among adults, older patients appear to be at greater risk for radiation-induced neural dysfunction [6]–[8]. It is not clear why WBI affects cognitive function or why aging may impact that neural response. Since all neural cell types probably are involved in the radiation response [9]–[11], it is critical to consider diverse cellular and molecular mechanisms. Studies to date indicate that changes in cell turnover and activation of neuroinflammatory pathways contribute significantly to neural deficits following WBI. These also may be processes where effects of normal aging and of WBI intersect [12].

WBI kills dividing cells and changes the proliferative potential of surviving progenitor cells. Reduced neurogenesis has been associated with cognitive deficits following WBI in young adult rodents [13]–[15] and, based largely on those studies, it has been proposed that conforming clinical brain irradiation to avoid neurogenic regions in patients may diminish or prevent cognitive deficits [16]–[18]. Significantly, however, there is no sustained effect of radiation on neurogenesis in older animals [12]. Moreover, WBI clearly alters functions of brain regions in which there is no adult neurogenesis [4], [19]–[23]. Thus, changes in neurogenesis likely contribute to neural deficits following WBI in the developing and young adult brain, but other mechanisms must contribute and may play a large role in older individuals.

In addition to reducing neurogenesis, brain irradiation can kill oligodendrocytes and alter glial turnover [9], [24]. Changes in white matter are the most common finding in imaging studies of patients suffering from WBI-induced cognitive changes, but cognitive dysfunction also may occur without evidence of white matter changes [25]–[27]. Most cycling cells in the normal adult brain produce oligodendrocytes or cells with the potential to become oligodendrocytes, and oligodendrocyte precursor cells (OPC) are found in both white- and gray matter [28]–[31]. Thus, radiation-induced death of oligodendrocytes and changes in OPCs could contribute to widespread deficits in neural signaling as both the maintenance and integrity of myelin and supporting functions of oligodendrocytes in gray matter become compromised [8], [32], [33]. How oligodendrocyte turnover changes during normal aging is not clear, however, and it is not known whether and how the vulnerability of oligodendrocytes and OPCs to radiation and their contribution to radiation-induced neural dysfunction may be altered in older individuals.

Among neural cells that contribute to neuroinflammation, microglia appear to be central to the development of radiation-induced injury [34]–[36]. Changes in microglia have been demonstrated repeatedly in the dentate gyrus (DG) of young adult animals examined at 1 week to 18 months after irradiation [12], [37]–[41]. Since most previous analysis of the microglial response has focused on the DG in developing and young adult animals, little is known about the neuroinflammatory response in regions outside of the DG, which lack neurogenesis and may respond differently to challenges. Similarly, little is known about the neuroinflammatory response to irradiation in the latter part of the lifespan (corresponding to the primary clinical population), despite evidence that both basal levels of neuroinflammatory markers and vulnerability to other pro-inflammatory stimuli clearly vary with age [12], [42]–[47]. Overall, it is likely that radiation-induced microglial responses and their sequelae may differ both regionally and between younger and older adults.

We previously examined the effects of radiation on neurogenesis, microglial density, and microglial expression of the rat homolog of CD68 (by immunolabeling with the ED1 antibody) in the subgranular zone (SGZ) of the DG [12]. This analysis demonstrated that in middle-aged and old animals, in contrast to young adults, WBI did not reduce neurogenesis below the level seen in age-matched control animals. A WBI-induced increase in proliferation of non-neurogenic cells was evident, particularly in older rats, but it was not clear what cell type(s) were involved.

To better understand the radiation-induced normal tissue response, we now have assessed the responses of non-neurogenic regions of gray matter and of white matter to a single 10 Gy dose of WBI. Previous experimental studies of mice [13], [14], [48] and rats [49], [50] demonstrated that a single dose of 10 Gy WBI produced cognitive deficits, supporting analysis of the response to single-dose WBI in rodents to assess neurobiological mechanisms of normal tissue injury. The analysis of gray matter in the present study compared responses of the CA1 and CA3 subfields of the hippocampus, since the two regions may be differentially susceptible to some aging-related and experimentally-induced changes [51]. The analysis of white matter assessed the corpus callosum (CC) as a representative region. In addition to assessing microglial density and ED1 labeling, we examined microglial morphology and quantified proliferation as additional indicators of microglial reactivity. We also analyzed effects of irradiation on the proliferation of non-neurogenic, non-microglial cells, which were identified as OPCs. The cellular markers were examined in rats irradiated at one of three adult ages and in age-matched, sham irradiated controls, in order to evaluate changes across the adult life span and assess aging-related changes in the response to WBI.

These analyses allowed us to test working hypotheses that i) non-neurogenic mechanisms, including but not limited to effects on other proliferating cells, can contribute to WBI-induced normal tissue damage in adults, and ii) that the nature and/or extent of these mechanisms differ among neural regions and are influenced by normal aging processes. The resultant evidence that gray- and white matter exhibit differential responses to aging and to WBI and that CA3 exhibits greater aging-related, and possibly WBI-induced, changes than CA1 indicate that future investigations of the mechanisms and treatment of radiation-induced normal tissue injury should include explicit assessment of region- and age-specific regulation of affected cell populations.

## Materials and Methods

Experimental procedures are summarized below; details not reported previously [12] are provided as supporting information (Text S1). Tissue processing was completed in cohorts with representation of each experimental condition and all quantitative analyses were completed blindly. All experimental endpoints were analyzed in all animals with Ns of 5–7 per experimental group.

### Ethics statement

This study was carried out in strict accordance with the recommendations in the Guide for the Care and Use of Laboratory Animals of the National Institutes of Health. All procedures were approved by the Institutional Animal Care and Use Committee of the Wake Forest School of Medicine (Protocol #A00-174). All irradiations were performed under ketamine/xylazine anesthesia and all efforts were made to minimize suffering. The animal facility at Wake Forest School of Medicine is accredited by the American Association for Accreditation of Laboratory Animal Care and complies with PHS-NIH and institutional policies and standards for laboratory animal care.

### Animals, irradiation procedures, and tissue processing

This study assessed 80 male Fischer 344×Brown Norway (F×BN) F_1_ hybrid rats obtained at 7, 17 or 27 months of age and acclimated for one month. Irradiated and sham control groups were anesthetized with ketamine/xylazine. Irradiated rats received a single, 10 Gy dose of WBI using a ^137^Cs irradiator. Rats (N = 5–7/group/age) were euthanized at 1 or 10 weeks after irradiation/anesthesia. One hemisphere from each rat was immersion-fixed and cryosectioned for immunolabeling.

### Immunohistochemistry and immunofluorescence

For analysis of microglial number, proliferation and phenotype, sections representing the entire anterior to posterior extent of the hippocampus were labeled by immunohistochemistry (IHC) [12] for the rat homolog of CD68 (ED1 antibody, commonly used as a marker of “activated” microglia or macrophages) or for Ki67 (dividing cells) and Iba1 (all macrophages/microglia). Additional sections were processed for multi-immunofluorescent (IF) labeling and confocal microscopy [52] using either: i) ED1 and Iba1, ii) anti-Ki67, anti-Iba1, and anti-chondroitin sulfate proteoglycan (NG2, commonly used as a marker of OPCs), or iii) NG2, Iba1, and platelet-derived growth factor receptor-alpha (PDGFR-a, another OPC marker).

### Stereological analyses

Iba1^+^ cells were counted in systematically random series (SRS) using the optical fractionator (Stereo Investigator, MBF Bioscience, Williston, VT); the average coefficient of error (Schmitz-Hof) was <0.05. Cells labeled by ED1 or Ki67 or double-labeled for Iba1 and Ki67 had low, heterogeneous densities and were counted in SRS using a modification of the optical disector [53], [54] and Neurolucida (MBF Bioscience) [42]. Counts of Iba1^+^, ED1^+^, Ki67^+^, and Ki67^+^/Iba1^+^ cells are expressed as # of cells/mm^3^. The percentages of “activated” or proliferating microglia were estimated by dividing the densities of ED1^+^ cells and of Iba1^+^/Ki67^+^ double labeled cells, respectively, by the density of Iba1^+^ cells in each region.

### Statistical analysis

In principle, cell counts for each ROI could be analyzed by three-way analysis of variance (ANOVA) with age, irradiation, and survival period as independent variables. Preliminary examination of the data indicated, however, that large radiation effects on some variables limited ability to detect changes associated with aging and that it was not feasible to conduct experiments with sufficient N and power to detect interactions in a three-way ANOVA. Therefore, statistical analyses were planned to answer three specific questions for each experimental endpoint. The threshold for significance was set at p<0.01 unless otherwise indicated. The first question was whether a given endpoint differed among sham irradiated, control animals of different ages and whether any effects of aging varied among ROIs. This was assessed by two-way ANOVA with age and region as main effects (measures from sham irradiated, control rats in the 1 week and 10 weeks survival cohorts within each age group were comparable and were combined for the analysis of regional and aging-related differences in baseline measures). The results of the ANOVAs are provided in Table 1. Where indicated, pair-wise comparisons were completed using the Holm-Sidak test and are reported in the relevant figures below. The second question was whether WBI significantly changed the value for each endpoint relative to sham irradiated animals of the same age. To test for these radiation effects, the means for each endpoint were compared between irradiated and age-matched, sham irradiated control rats using two-sample, two-tailed t tests with the threshold for significance Bonferroni corrected to p<0.006 (3 ages×3 regions = 9 comparisons, p<0.05/9 = 0.006). Finally, to determine whether the magnitude of radiation effects on each endpoint differed among regions and/or among ages, difference values (irradiated/mean control value) were calculated and compared by two-way ANOVA with region and age as main effects and using Holm-Sidak post hoc tests for pair-wise comparisons. The results of the ANOVAs are provided in Table 2 and the results of pair-wise comparisons are reported in the relevant figures below. For analysis of radiation effects, separate analyses were completed for the 1 week and 10 week survival groups. Statistical analyses were completed using Sigmastat (SYSTAT Software, San Jose, CA).

**Table 1:** Results of two-way ANOVAs testing for effects of age and region on baseline measures of each dependent variable in sham irradiated, control rats (results of post-hoc tests provided in Figures 2, 3, 6 and 7).

	Age	Region	Interaction
#Iba1	*F(2,90) = 13.20 p<0.001*	*F(2,90) = 29.22, p<0.001*	*F(4,90) = 8.06, p<0.001*
#Ki67^+^/Iba1^+^	*F(2,89) = 19.01, p<0.001*	*F(2,89) = 15.62, p<0.001*	*F(4,89) = 7.76, p<0.001*
%Iba1^+^/Ki67^+^	*F(2,88) = 10.00, p<0.001*	*F(2,88) = 8.78, p<0.001*	*F(4,88) = 2.60, p = 0.04*
#ED1^+^	*F(2,85) = 113.51, p<0.001*	*F(2,85) = 218.18, p<0.001*	*F(4,85) = 49.15, p<0.001*
Est. %ED1^+^	*F(2,84) = 42.01, p<0.001*	*F(2,84) = 115.78, p<0.001*	*F(4,84) = 7.27, p<0.001*
#Ki67^+^/Iba1^−^	*F(2,86) = 1.62, p = 0.20*	*F(2,86) = 364.30, p<0.001*	*F(4,86) = 1.01, p = 0.48*

**Table 2:** Results of two-way ANOVAs testing for effects of age and region on the WBI-induced change in each dependent variable (results of post-hoc tests provided in Figures 2, 3, 6 and 7).

		Age	Region	Interaction
#Iba1	1 week	*F(2,42) = 1.55, p = 0.22*	*F(2,42) = 1.06, p = 0.36*	*F(4,42) = 0.28, p = 0.89*
	10 weeks	*F(2,38) = 0.94, p = 0.40*	*F(2, 38) = 0.82, p = 0.45*	*F(4, 38) = 0.45, p = 0.77*
#Ki67^+^/Iba1^+^	1 week	*F(2,41) = 3.61, p = 0.04*	*F(2,41) = 22.61, p<0.001*	*F(4,41) = 4.23, p = 0.006*
	10 weeks	*F(2,36) = 19.26, p<0.001*	*F(2,36) = 21.70, p<0.001*	*F(4,36) = 4.11, p = 0.008*
%Iba1^+^/Ki67^+^	1 week	*F(2,40) = 5.66, p = 0.007*	*F(2,40) = 17.29, p<0.001*	*F(4,40) = 5.36, p = 0.001*
	10 weeks	*F(2,37) = 21.87, p<0.001*	*F(2,37) = 24.62, p<0.001*	*F(4,37) = 4.74, p = 0.003*
#ED1^+^	1 week	*F(2,40) = 9.00, p<0.001*	*F(2,40) = 26.96, p<0.001*	*F(4,40) = 1.63, p = 0.19*
	10 weeks	*F(2,36) = 8.30, p = 0.001*	*F(2,36) = 3.85, p = 0.03*	*F(4,36) = 1.40, p = 0.26*
Est. %ED1^+^	1 week	*F(2,38) = 8.09, p<0.001*	*F(2, 38) = 17.00, p<0.001*	*F(4, 38) = 1.02, p = 0.41*
	10 weeks	*F(2,36) = 5.46, p = 0.008*	*F(2,36) = 2.03, p = 0.15*	*F(4,36) = 1.323, p = 0.28*
#Ki67^+^/Iba1^−^	1 week	*F(2,41) = 5.07, p = 0.01*	*F(2,41) = 74.17, p<0.001*	*F(4,41) = 3.38, p = 0.02*
	10 weeks	*F(2,37) = 5.45, p = 0.008*	*F(2,37) = 6.57, p = 0.004*	*F(4,37) = 0.49, p = 0.75*

## Results

### Microglial morphology

To simplify presentation, the Iba1^+^ (and ED1^+^) cells analyzed in this study are referred to as microglia. Possible contributions of peripherally-derived macrophages or other bone-marrow derived cells (BMDC) are considered in the Discussion.

**Figure 1:**
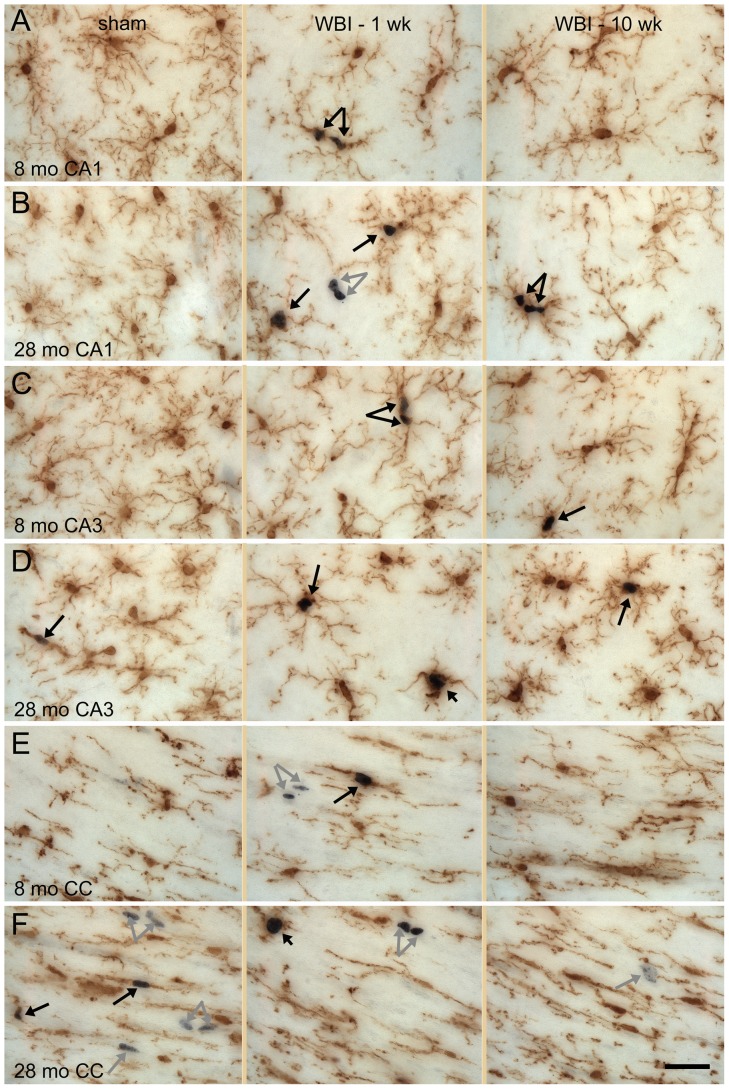
Microglial morphology. Immunolabeling for Iba1 (brown) demonstrates microglia in CA1 (A, B), CA3 (C, D) and CC (E, F). The appearance of microglia in sham controls and at 1 and 10 weeks after WBI is illustrated for young adult (8 mo) and old (28 mo) rats. Double-labeling for Iba1 and Ki67 (black) demonstrated proliferation of microglia (black arrows) and of non-microglial cells (gray arrows). Most proliferating microglia had extensive processes (long arrows) but a few (short arrows in D and F WBI-1 wk) exhibited larger cell bodies and only a few short processes. Scale bar = 25 µm. Images shown are z projections of five image planes representing 10 µm section depth.

Iba1^+^ cells in CA1, CA3, and other regions of gray matter had round or oval cell bodies and ramified processes that extended relatively symmetrically from the cell body to cover areas approximately 50 µm in diameter (Fig. 1A through D, left panels). The territories occupied by individual microglia typically were separated by a few micrometers. Regardless of age or irradiation status, most microglia bore numerous branched processes. The morphology often described as “activated” - macrophage-like cells with enlarged cell bodies and few processes - that is common following challenges that induce significant neuronal death [55] was rare, although a few such cells were evident in the oldest and irradiated animals (Fig. 1D and F, middle panels). Following WBI, an increased proportion of Iba1^+^ cells in gray matter had processes that were somewhat shorter and thicker and the regions separating microglial territories appeared expanded (Fig. 1). This qualitative impression was supported by quantitative analysis of the areas not occupied by microglia or their processes (see Supplemental Materials and Methods and Fig. S1 and S2). There appeared to be a similar but more modest increase in the area not occupied by microglia when comparing old to young adult sham rats; quantitative analysis revealed a significant increase in middle aged and old rats in CA1 but not in CA3 (Fig. S2).

Iba1^+^ cells in the white matter typically bore primary processes oriented in the direction of the axon bundles and finer processes oriented orthogonally (Fig. 1E, left panel). Qualitatively, the area occupied by microglia and their processes in the CC (Fig. 1F, left panel) appeared greater in old, sham irradiated control rats compared to middle-age and younger rats. This was confirmed by quantitative analysis (Fig. S2). In response to WBI, the representation of microglia in the CC appeared to regress slightly at one week after WBI (compare middle to left panels in Fig. 1E and F). Quantitative analysis revealed that the WBI-induced change was statistically significant only in young adult rats and that the change was smaller in the CC than in the hippocampal gray matter (Fig. S2).

### Microglial density

**Figure 2:**
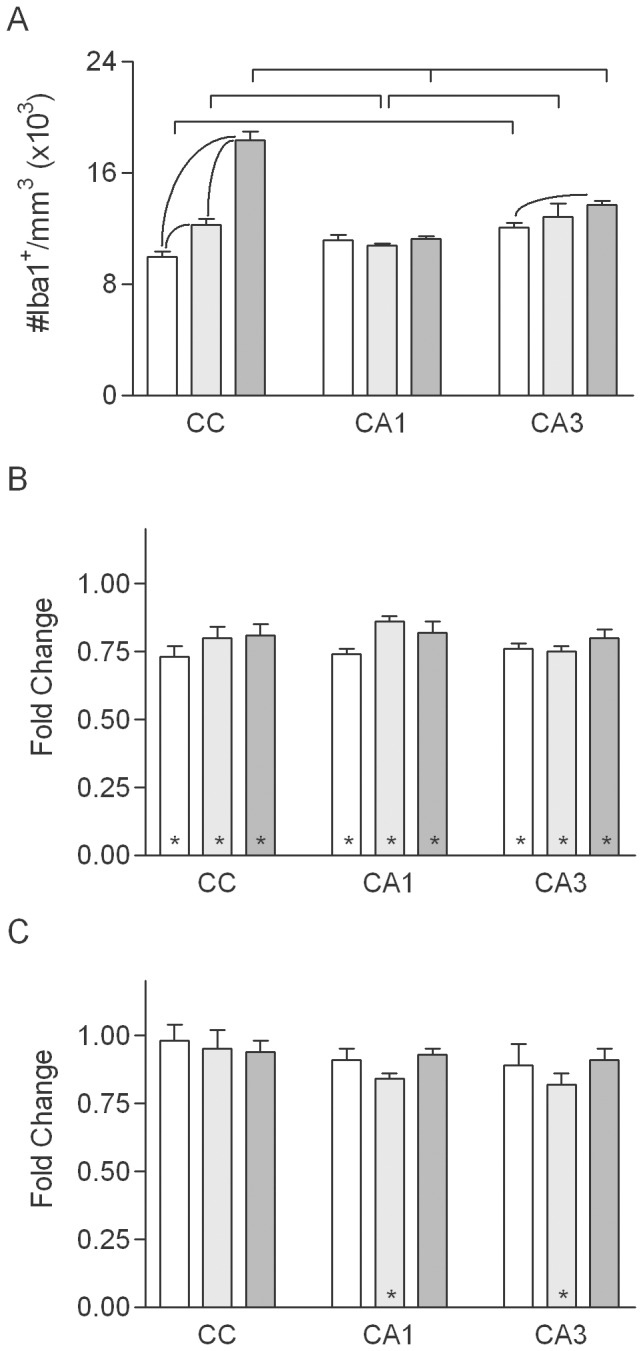
Density of microglia. A. Microglial density in normally aging rats in the young adult (open bars), middle-aged (light gray bars) and old (dark gray bars) groups (values combined for sham irradiated, control rats of each age from the 1- and 10-week survival groups. Mean values (+sem) are indicated. Significant effects of age within each region are indicated within the group of bars representing that region; significant differences among regions at each age are indicated above the bars. B, C. WBI-induced changes in microglial density at 1 and 10 weeks (respectively) after WBI. Asterisks indicate that the mean for irradiated animals was significantly different from that for age-matched, sham irradiated controls. There were no significant effects of region or age on the magnitude of the WBI-induced changes in density.

Stereological counts of Iba1^+^ cells in sham-irradiated, control animals (Fig. 2) demonstrated that the density of microglia did not vary with age in CA1 and increased slightly in the oldest rats in CA3, whereas the density in the CC was higher in middle-aged than young adult rats and higher still in old rats (∼two-fold greater in old versus young adult). Regardless of age at time of irradiation, WBI decreased the microglial density in CA1 and CA3 by ∼20–25% at 1 week post-irradiation (Fig. 2B). Microglial densities after WBI appeared more variable in the CC than in the hippocampus. Microglial densities were at or near control levels by 10 weeks post-WBI (Fig. 2C) but remained significantly reduced (∼15%) in CA1 and CA3 of middle-aged rats. Thus, irradiation of the adult brain leads to microglial death, as previously shown in developing rats [56].

### Microglial proliferation

Proliferating microglia were identified by the presence of Ki67^+^ nuclei surrounded by Iba1^+^ cytoplasm (resolved in IHC-labeled sections by focusing through with a high numerical aperture, 60× objective). Labeling of individual cells for Ki67 and Iba1 was confirmed in sections double-labeled for Iba1 and Ki67 by IF and examined by confocal microscopy (see below). Qualitative assessment revealed a very small population of proliferating microglia in sham irradiated animals and a clear increase in irradiated rats (Fig. 1). In the latter, Ki67^+^/Iba1^+^ cells often appeared in pairs or clusters (e.g., Fig. 1A, B and C). Pairs of closely adjacent Iba1^+^ cell bodies that were not Ki67^+^ also were common; this likely reflected recent, radiation-induced cell division since such closely apposed microglial cell bodies were not seen in sham irradiated controls. Individual cells containing two Ki67^+^ nuclei, apparently cells undergoing cytokinesis at the time of fixation, also were common in irradiated rats. A wide range of microglial morphologies was evident among the dividing microglia and most bore extensively ramified processes (Fig. 1A–D). Only a few of the dividing Iba1^+^ cells had the large cell body and restricted processes associated with “activated” microglia.

**Figure 3:**
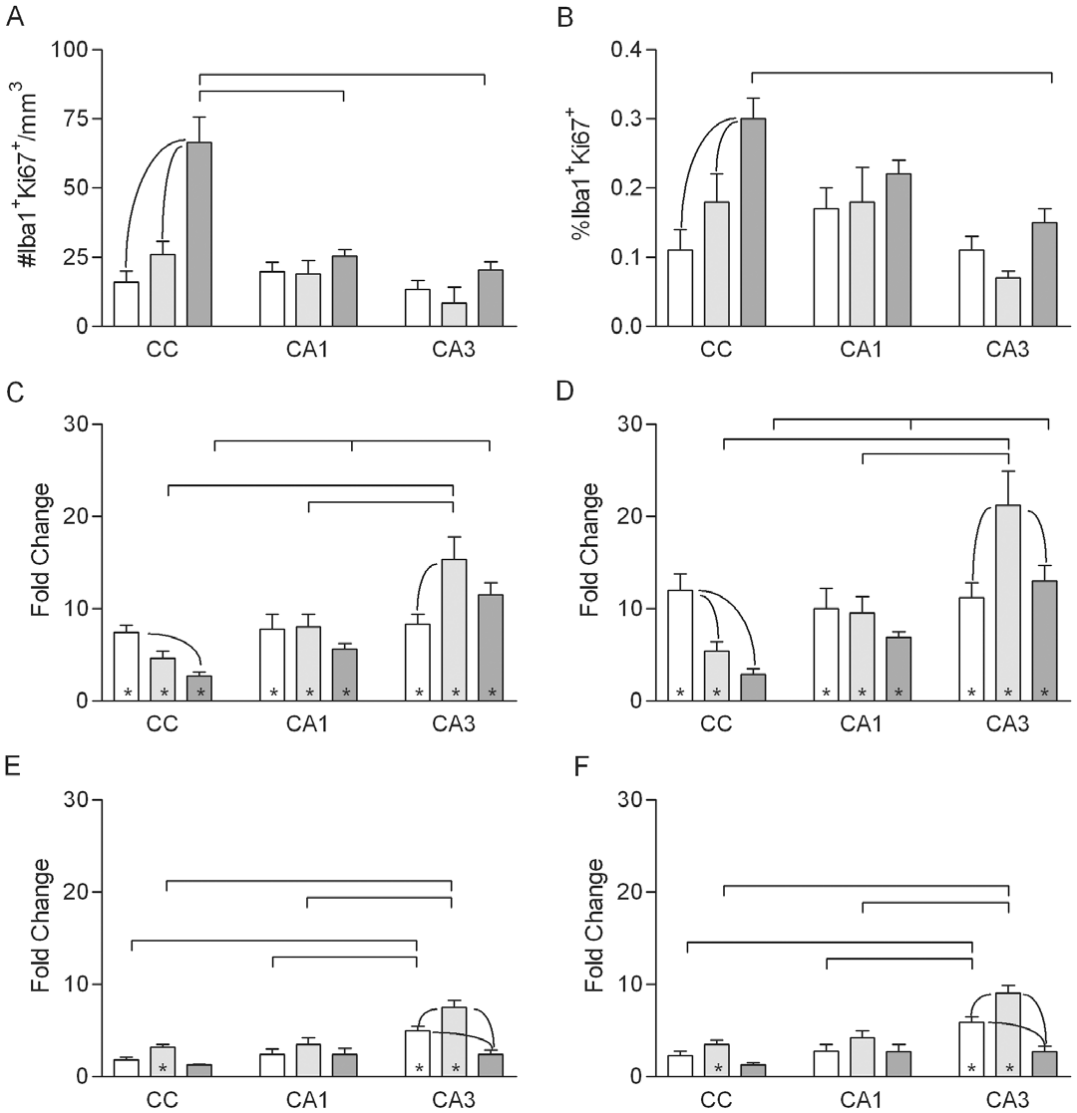
Density and fraction of proliferating microglia. A, B. Density and percentage, respectively, of Iba1^+^ cells expressing Ki-67 in normally aging rats in the young adult (open bars), middle-aged (light gray bars) and old (dark gray bars) groups (values combined for sham irradiated, control rats of each age from the 1- and 10-week survival groups). Mean values (+sem) are indicated. Results of post-hoc comparisons are indicated as described for Figure 2. C–F. WBI-induced changes in the density and percentage of Iba1^+^ cells expressing Ki-67 at 1 (C, D) and 10 (E, F) weeks after WBI. Asterisks indicate that the mean for irradiated animals was significantly different from that for age-matched, sham irradiated controls.

In sham irradiated, control rats the densities of proliferating microglia were similar in CA1 and CA3 and did not differ significantly among the three ages (Fig. 3A). The density of proliferating microglia in the CC was similar to that in CA1 and CA3 in young, sham irradiated animals, but in old rats the density in the CC was approximately three-fold greater than that in the CC of young adults or in CA1 or CA3 at any age.

In young adult rats at 1 week post-irradiation, WBI increased proliferating microglia approximately eight-fold in CA1, CA3 and the CC, with no significant differences among the three regions (Fig. 3C). Aging altered the radiation response, and the combined effects of aging and WBI varied among neural regions. In the hippocampus, the WBI-induced increase in microglial proliferation was stable with age in CA1 and increased approximately two-fold with age in CA3, being highest in middle-aged rats. In contrast, the radiation-induced increase in microglial proliferation in the CC was smaller in older rats than in young adults (Fig. 3C). Although effects of WBI on the density of Ki67^+^/Iba1^+^ cells were reduced by 10 weeks post-irradiation (Fig. 3E), microglial proliferation remaining significantly elevated in CA3 of young adult rats and in CA3 and the CC of middle-aged rats.

Since both the overall density of microglia and the density of proliferating microglia were affected by normal aging and WBI (in opposite directions in the case of WBI), we estimated the percentage of microglia that were proliferating under each condition (density of Ki67^+^/Iba1^+^ cells divided by the density of Iba1^+^ cells). In sham irradiated, control rats, dividing microglia represented 0.1 to 0.2% of the population in CA1 and CA3 at all ages; the percentage was similar in the CC of young adult and middle-aged rats but was approximately three-fold higher in the old animals (Fig. 3B). Overall, the patterns of aging-related and WBI-induced changes in the percentage of proliferating microglia paralleled the changes seen in the density (Fig. 3D, F).

### Microglial expression of CD68

**Figure 4:**
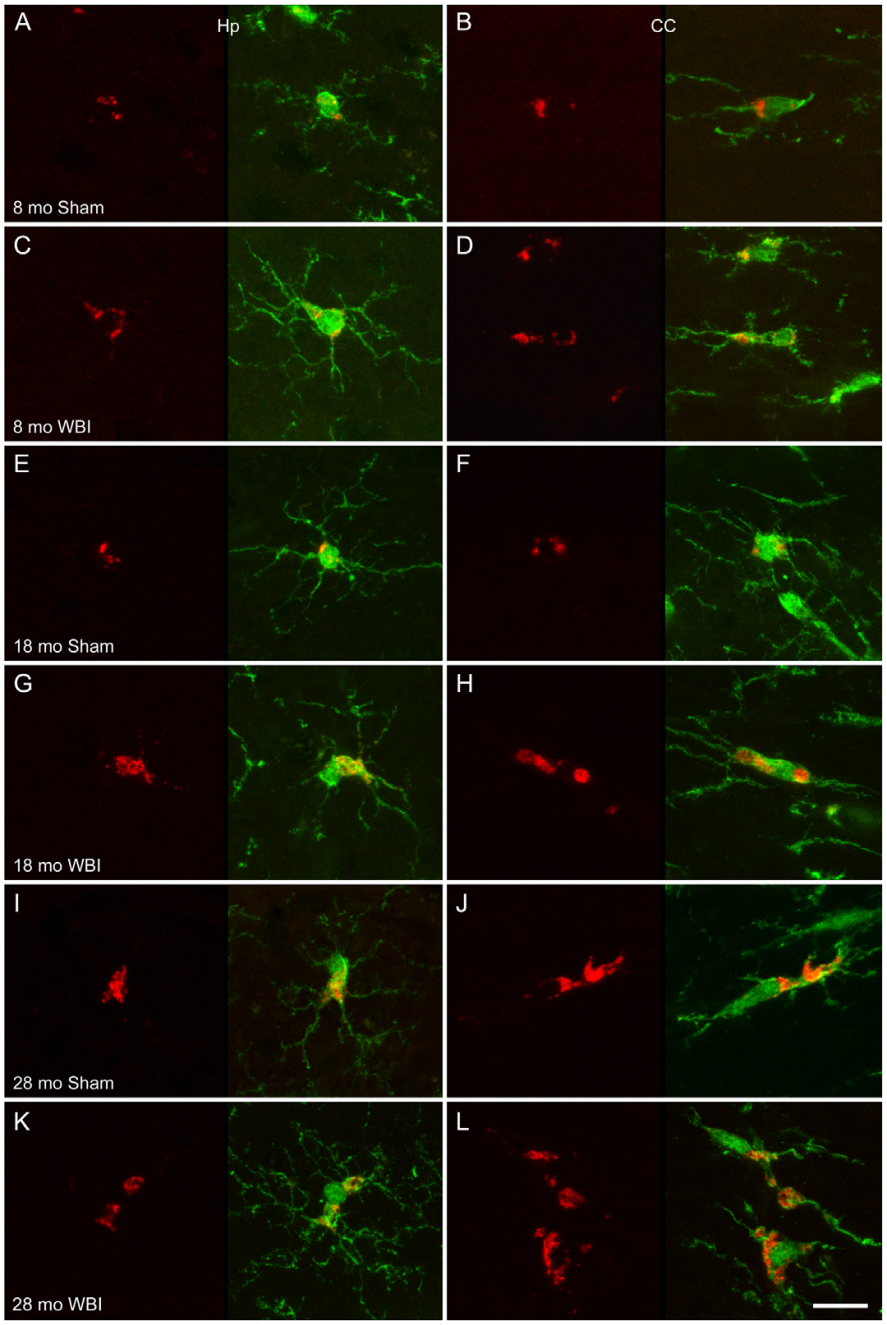
Morphology of ED1^+^ microglia. Representative examples of double-IF labeling with ED1 (red) and anti-Iba1 (green) are shown from animals of the indicated ages and conditions. For each example an image stack was collected (Z step = 0.5 or 1 µm) and projected to a single image plane (maximum intensity function; left panel ED1 only, right panel merged image). Examples from the hippocampus (Hp, CA1 or CA3) are shown on the left and from the CC on the right. ED1 labeling was most common in the perinuclear region but also appeared in enlarged regions of the cell body (e.g., as in D and G–L) and sometimes within cell processes (e.g., upper cell in L). Intensity levels in some images were adjusted to optimize demonstration of the location of ED1 labeling and the morphology of Iba1^+^ cells; therefore, differences in brightness do not necessarily reflect differences in labeling intensity. Scale bar = 15 µm.

Confocal analysis of sections labeled by double immunofluorescence confirmed that ED1 labeling was restricted to Iba1^+^ cells. The majority of ED1^+^ cells were highly ramified (Fig. 4). ED1 labeling was most common in cell bodies but also appeared in some processes (e.g., Fig. 4D, J, L).

**Figure 5:**
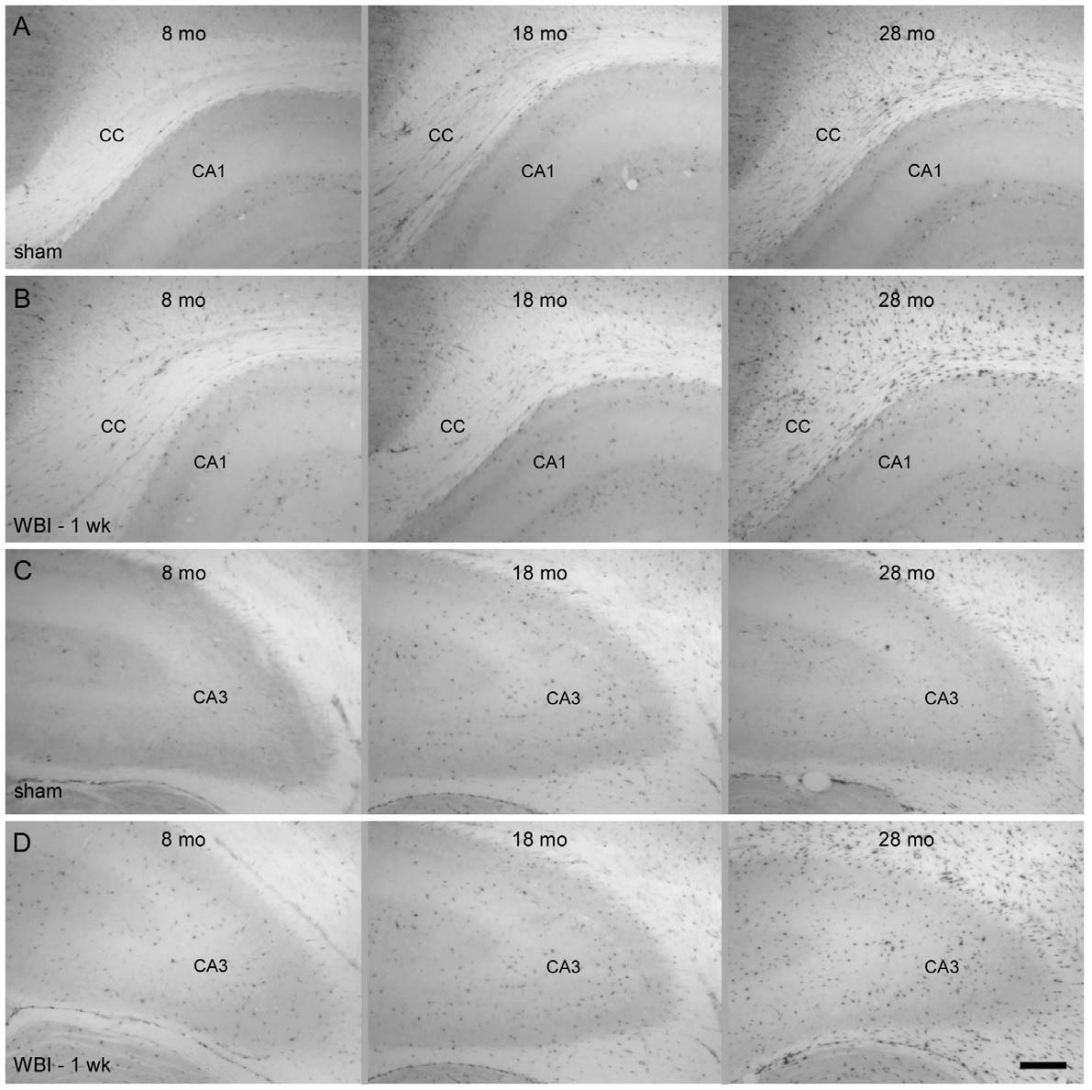
IHC labeling with ED1. Images illustrating ED1 labeling in the CC and CA1 (A, B) and CA3 (C, D) of young adult, middle-aged and old rats following sham irradiation (A, C) or at 1 week after WBI (B, D), Scale bar = 250 µm.

**Figure 6:**
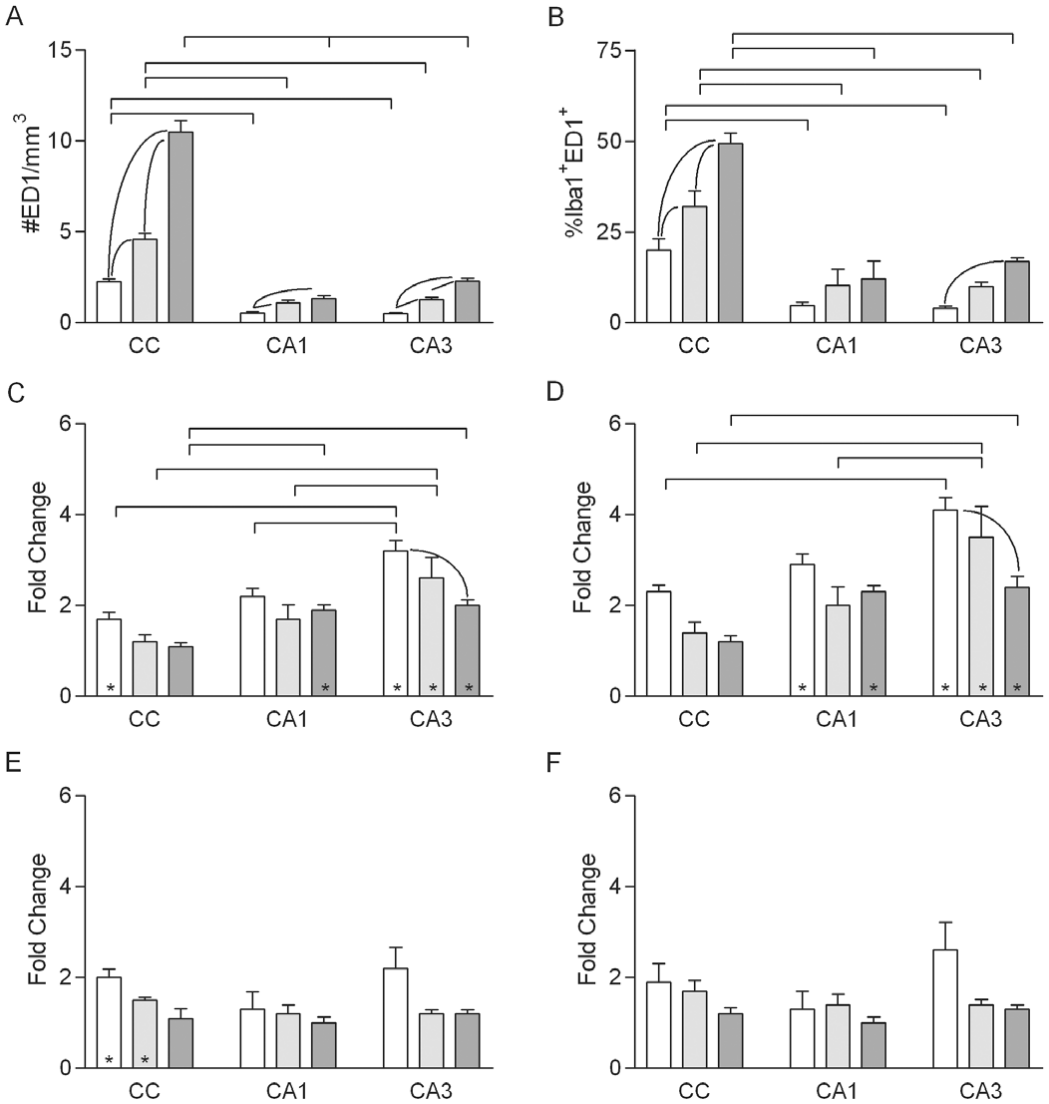
Density and fraction of ED1^+^ microglia. A, B. Density of ED1^+^ cells (A) and estimated percentage of Iba1^+^ cells expressing ED1 antigen (B) in normally aging rats in the young adult (open bars), middle-aged (light gray bars) and old (dark gray bars) groups (values combined for sham irradiated, control rats of each age from the 1- and 10-week survival groups). Mean values (+sem) are indicated. Results of post-hoc comparisons are indicated as described for Figure 2. C–F. WBI-induced changes in the density and percentage of ED1^+^ microglia at 1 (C, D) and 10 (E, F) weeks after WBI. Mean values (+sem) are indicated. Asterisks indicate that the mean for irradiated animals was significantly different from that for age-matched, sham irradiated controls.

In sham irradiated, control rats, the density and percentage of IHC-labeled ED1^+^ microglia (Fig. 5) were higher in older animals than in young adults (Fig. 6A, B). In CA1, ED1^+^ cells increased between young- and middle-age but not between middle- and old age. In CA3, the density of ED1-labeled cells increased between young- and middle age and further between middle- and old age. The density and percentage of ED1^+^microglia were substantially higher in the CC than in CA1 or CA3 at all ages and also increased more with age; in the oldest, sham-irradiated animals 50% of microglia in the CC were ED1^+^.

At 1 week post-irradiation, WBI increased the density and percentage of ED1^+^ cells in CA1 of old rats and in CA3 at all ages (Fig. 6C, D). The WBI-induced increase was significantly greater in CA3 than in CA1 in young adult and middle-aged animals. Apparent increases in activated microglia following irradiation were smaller in the CC than in CA1 and CA3 and measures were more variable, such that the trend toward radiation-induced changes in the CC reached statistical significance only in young rats. By 10 weeks post-irradiation (Fig. 6E, F), the density and percentage of ED1^+^ cells largely returned to age-matched, sham irradiated, control levels (a small increase in density in the CC of young adult and middle-aged rats was not significant when expressed as the percentage of microglia).

### Proliferation of oligodendrocyte precursor cells

**Figure 7:**
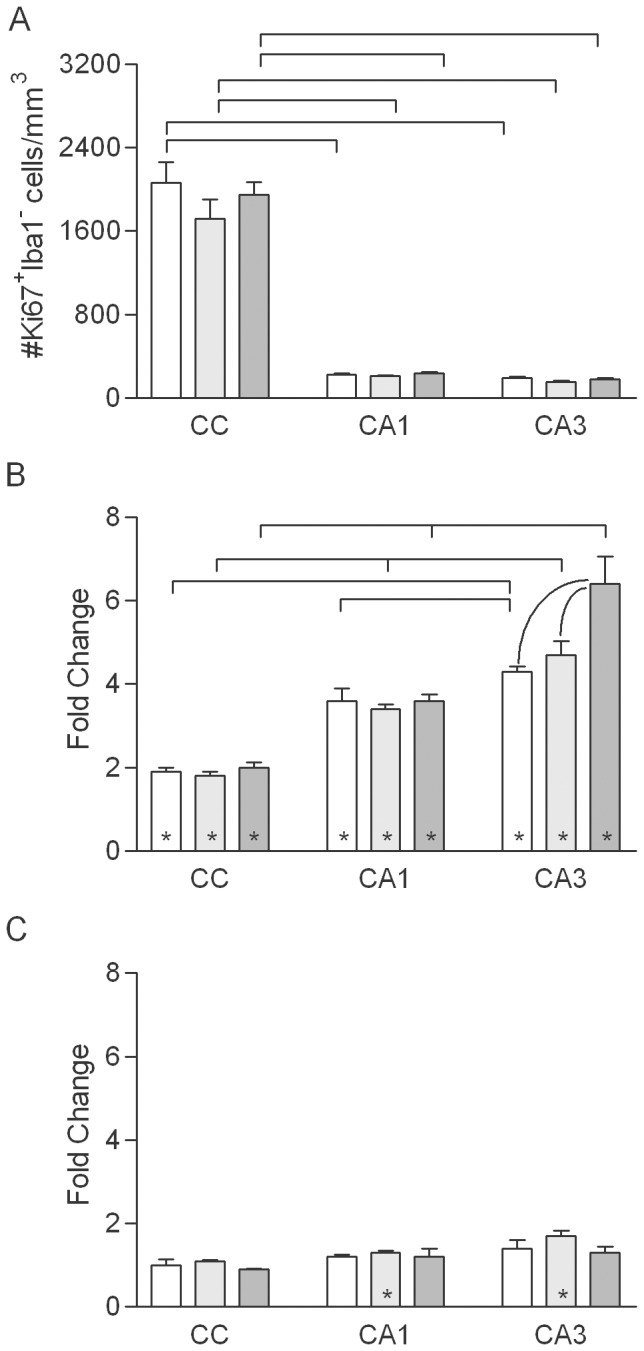
Density of proliferating non-microglial cells. A. Density of Ki67^+^ cells that did not express Iba1 in normally aging rats (values combined for sham irradiated, control rats of each age from the 1- and 10-week survival groups). Mean values (+sem) are indicated. Results of post-hoc comparisons are indicated as described for Figure 2. B, C. WBI-induced changes in the density of Ki67^+^/Iba1^−^ cells at 1 (B) and 10 (C) weeks after WBI. Mean values (+sem) are indicated. Asterisks indicate that the mean for irradiated animals was significantly different from that for age-matched, sham irradiated controls.

**Figure 8:**
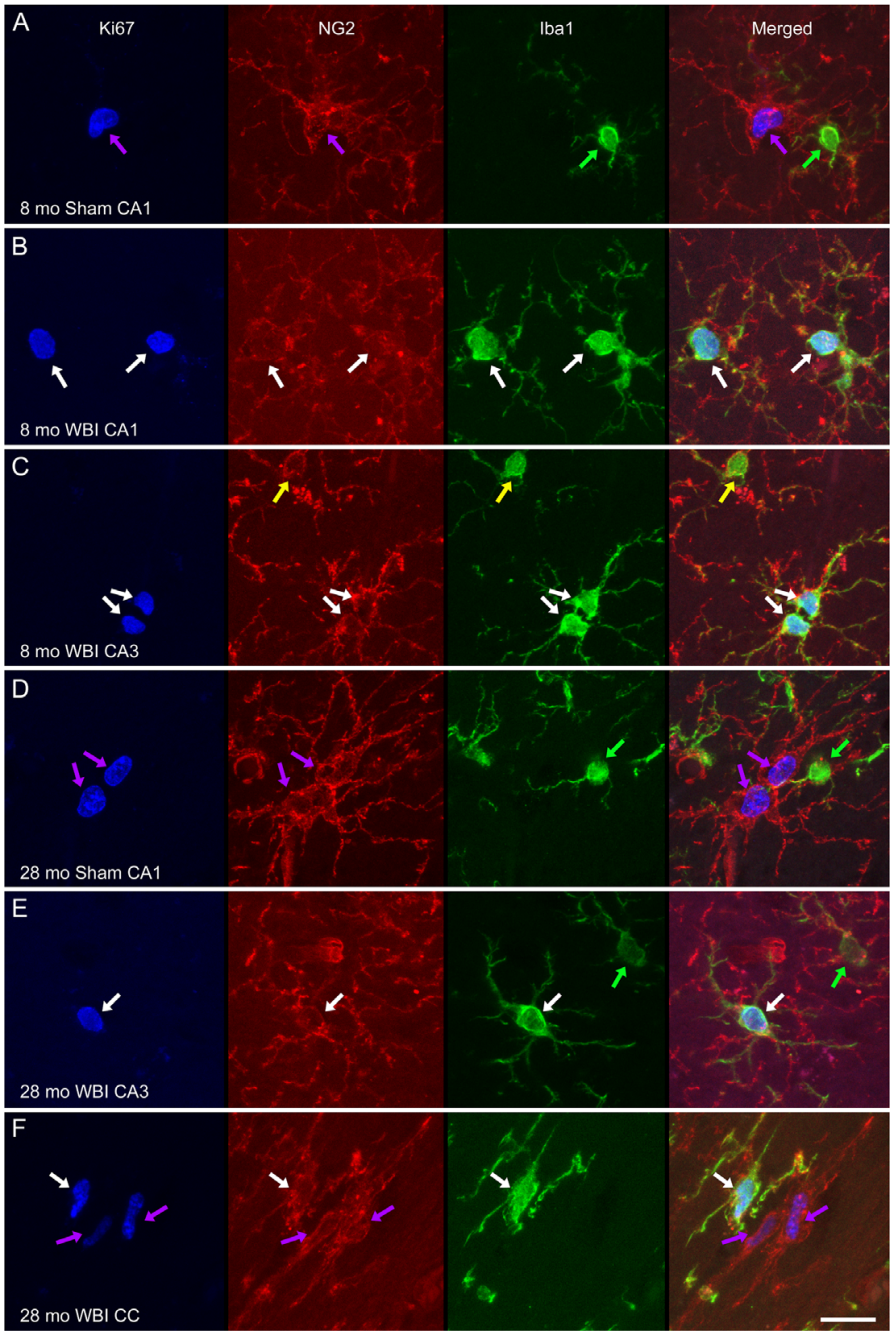
Phenotype analysis of proliferating non-microglial cells. Representative examples of triple-IF labeling for Ki67 (blue), NG2 (red), and Iba1 (green) are shown from animals of the indicated ages and conditions. For each example an image stack was collected (Z step = 0.5 µm) and projected to a single image plane (maximum intensity function). Dividing microglia (Ki67^+^/Iba1^+^/NG2^+^) are indicated by white arrows. Among the Iba1^+^ microglia that were not dividing, some co-expressed NG2 (yellow arrows) while others did not (green arrows), although NG2^+^ process often were located in close approximation to the latter. The dividing cells that did not express Iba1 were NG2^+^ (purple arrows). Scale bar = 15 µm.

Dividing (Ki67^+^) cells that did not express Iba1 were apparent in all regions of the brain (examples of such cells in the hippocampus and CC are shown in Fig. 1B, E and F). In sham irradiated control rats, the densities of Ki67^+^/Iba1^−^ cells did not differ among the three ages but, regardless of age, the density was approximately 10-fold higher in the CC than in CA1 and CA3 (Fig. 7A). The higher density in the CC was consistent with the expectation that proliferating cells that were not Iba1^+^ represented primarily OPCs. To further characterize the phenotype of Ki67^+^/Iba1^−^ cells, 500 Ki67^+^ cells (from two animals each in the young and old sham and young and old 1 week WBI groups) were examined in sections triple-labeled for Ki-67, Iba1, and the OPC marker NG2 (Fig. 8). Virtually every Ki67^+^ cell that did not express Iba1 did express NG2 (one Ki67^+^ cell expressed neither NG2 nor Iba1). Consistent with counts in IHC-labeled sections, Ki67^+^/NG2^+^/Iba1^−^ cells (presumptive OPCs) were more abundant in the CC than in the hippocampus. Examination of more than 400 NG2^+^ cells in sections that were triple-labeled for Iba1, NG2, and a second marker of OPCs, PDGFR-a revealed that all but three NG2^+^ cells that did not express Iba1 did express PDGFR-a (data not shown).

At 1 week after WBI, the density of Ki67^+^/Iba1^−^ cells increased in each region and at each age (Fig. 7B). The relative increase in Ki67^+^/Iba1^−^ cells at 1 week after WBI was greater in CA3 (6-fold) and CA1 (3-fold) than in the CC (2-fold), although the absolute increase was greater in the CC (increase approximately 2500 cells/mm^3^ in the CC versus <700/mm^3^ in CA1 or CA3) since the density of dividing OPC was much higher in white matter. At 10 weeks after WBI (Fig. 7C), the densities of Ki67^+^/Iba1^−^ cells remained slightly but significantly elevated (25–65%) in CA1 and CA3 of middle-aged rats.

### Regional volume analysis

Estimates of regional volumes obtained with counts of Iba1^+^ and Ki67^+^/Iba1^+^ cells were analyzed to assess whether changes in volume could account for or contributed to changes in the densities of labeled cells. Two-way ANOVA testing for differences among regions and ages revealed no effect of age on regional volumes (*F(2, 85) = 0.510, p = 0.60*). Similarly, comparing volume estimates from sham-irradiated and irradiated rats of each age and at each survival point revealed no effect of WBI on the volume of the CC, CA1 or CA3 (all p>0.13 by two-tailed t tests).

## Discussion

Radiation-induced neural deficits appear to be exacerbated in older compared to young adult patients [6]–[8] and many functional changes that develop following WBI (e.g., changes in memory, attention, and executive function) [32] resemble those that often occur normally late in the lifespan. This suggests common cell types and mechanisms may contribute to both aging-related and WBI-induced cognitive changes and that aging and radiation-induced normal tissue injury may interact in ways that influence clinical outcomes. The general goals of this study were to assess whether and how microglia and OPCs, cell types thought to be critical to the neurobiological response to clinical irradiation, differ regionally and among younger and older adults and to test for regional and aging-related differences in the responses of these cells.

### Microglial responses

The current understanding of microglial responses to neural damage comes largely from insults (e.g., bacterial toxins, ischemia, trauma) that induce acute neuronal death, recruit peripheral immune cells, and cause resident microglia to transform into phagocytic, macrophage-like cells. The robust response to such challenges initially led to the idea that microglia switch between a resting state and an “activated”, phagocytic, macrophage-like state. There is increasing recognition, however, that microglia can adopt a wide range of effector phenotypes with elements that may damage *or* support other cells following a challenge [57], [58]. The present study permitted assessment of microglial responses under pro-inflammatory conditions, aging and moderate-dose WBI, that are relatively moderate but nevertheless associated with cognitive changes. Multiple cognitive deficits have been demonstrated previously in middle-aged and older rats, and WBI at 10 Gy produces cognitive deficits in rodents (see Introduction). WBI at 10 Gy does not, however, kill mature neurons [9], [59], nor does it appear to result in recruitment of circulating monocytes to differentiate as parenchymal macrophages. We could not definitively differentiate microglia from peripheral macrophages in the present study, since Iba1, ED1 and other commonly used markers label both. One recent experiment using parabiotic, chimeric animals demonstrated that a 11 Gy dose of irradiation to the brain was not sufficient to result in migration of macrophage precursors from the periphery into the CNS in young adult rodents [60], consistent with the conclusion that the Iba1^+^ cells examined here were primarily or exclusively microglia. A possible contribution of peripherally derived cells remains possible, however, since another study using bone marrow reconstitution with bone marrow cells harvested from GFP+ mice revealed that some type(s) of BMDC contributed to the microglial population following irradiation, although not to the population of proliferating microglia [61].

The recognition that stimulated microglia can exhibit a range of phenotypic responses (not just switch between a resting state and an “activated” state), raises questions about the relationships among different responses within individual cells. In the present study, most ED1^+^ and/or dividing microglia, even those undergoing cytokinesis, bore extensive processes and exhibited morphologies as varied as the microglial population as a whole. This morphological diversity of the ED1^+^ and proliferative populations indicates that microglia can divide and increase expression of CD68 without developing a macrophage-like morphology. In the absence of profound damage and extensive neuronal death, there is no transition to an “activated” phenotype, but rather an array of structural and functional changes that are regulated to some extent independently.

#### Microglial responses to normal aging

The results of the present study add to previous reports that microglia are altered during normal aging and, moreover, demonstrate that the extent and nature of changes differs among neural regions. Microglia are primarily local effectors, so their influence depends first on their regional density, which represents a balance of proliferation, death, and migration. At least some of these processes differ among neural regions and across the adult lifespan. Microglial density is stable in the DG [12] and CA1 during aging, but there is a modest (10–15%) increase in CA3 and a much larger (two-fold) increase in the CC (present study). The increase in white matter is associated with increased microglial proliferation. Like the size of the population and the extent of proliferation, expression of the lysosomal and cell-surface antigen CD68 (recognized by the ED1 antibody and used widely as a marker of microglial response) [40], [41], [62], [63] varies among regions and across ages. Regardless of age, the density and percentage of ED1^+^ microglia are much higher in the CC than in the hippocampal gray matter. As for microglial density and turnover, aging-related regulation of CD68 differs between CA1 and CA3 and between the hippocampus and CC. The origins and functional impact of the aging-related and regionally-dependent microglial changes remain to be established. The observation that aging-related microglial changes are greater in CA3 than in CA1 is consistent, however, with reports that other aging-related structural and functional changes are greater in CA3 [51]. The larger microglial population within the CC (compared to gray matter) and the substantial response of that population to aging suggests the microglial population responds to and plays a role in ongoing myelin turnover, which appears to be increased in older animals [64], [65].

#### Microglial responses to WBI

The regional and aging-related differences in microglial density, microglial proliferation, and CD68 expression led us to assess how these differences in the basal state of the microglial population affect the response to the pro-inflammatory challenge of WBI. A variety of evidence indicated that responses to WBI might differ among regions and ages, but the nature of any differences was difficult to predict. As discussed below, some studies suggest that regions/ages characterized by higher levels of microglial activity would show greater responses to WBI, whereas other studies suggest those regions/ages would show reduced responses.

There are several reports that a modest inflammatory stimulus can amplify the response of microglia to a subsequent stimulus. For example, when microglia are exposed to an inflammatory stimulus *in vitro* they exhibit greater up-regulation of pro-inflammatory gene expression following irradiation, compared to microglia without pre-stimulation [66]. In addition, *in vivo*, microglial proliferation and CD68 expression induced by intracerebral hemorrhage are greater in older rats than in young adults [67]. Thus, aging and/or focal differences could “prime” microglia such that there is a greater response to WBI in regions and at ages characterized by higher basal levels of microglial density, proliferation, and CD68 expression. In the present study, there was only limited evidence for an amplified response to WBI in regions characterized by higher basal levels of the microglial markers. By multiple characteristics, CA3 exhibited a greater response than CA1 to aging and to WBI and also was the region most likely to still show a significant radiation response at 10 weeks after WBI. In contrast, however, radiation-induced changes in the microglial markers examined were not amplified in white matter compared to gray matter and were not greater in old rats than in young adults.

One also might reasonably predict a smaller response to WBI in regions and at ages characterized by higher basal levels of microglial activity, since higher basal levels of activity might permit microglia to repair damage from a modest, acute challenge like moderate-dose WBI with little or no additional up-regulation. Consistent with that view, the patterns of radiation responses in the present study (other than the greater responsiveness of CA3) appeared to be inversely related to regional and aging-related patterns in basal levels of microglial markers. That is, regions and ages with higher basal levels showed less relative increase following WBI.

Rather than indicating a smaller need for up-regulation, the diminished radiation response in older rats may reflect a reduced *ability* of microglia to respond, at least with respect to some phenotypic changes. Streit and colleagues have presented evidence that microglia in the human brain undergo cellular senescence during aging [68]. Specifically, aging-related replicative senescence may limit the ability of microglia to proliferate in response to challenges. Changes in cellular structure, including loss of ramification and the presence of cytosolic inclusions and spheroid swellings in processes, may reflect dystrophic microglia with diminished functional capacities [69]. Whether microglia in the aging rodent brain undergo similar senescence has not been clear. The data presented here indicate that they may but with respect to only some aspects of their response to inflammatory challenge. The structure of Iba1-labeled microglia in this study (Fig. 1) did not change significantly over the ages examined, consistent with a previous report that the aging-related breakdown in microglial morphology described in the human brain does not occur in rodents [70]. Similarly, the density of microglia and the level of ongoing microglial proliferation in control rats were sustained in the hippocampus and increased substantially in the CC across the adult lifespan, so there is no indication of replicative senescence under basal conditions. The proliferative burst in response to WBI did decrease in the CC of old compared to young adult rats, suggesting there may be an aging-related decline in replicative capacity in response to an acute challenge. No such decline was evident in the hippocampus, however, and the response actually increased with age in CA3 (at least through middle age). Overall, there clearly is dynamic regulation of the size of the microglial population in response to a challenge even in the oldest rats. With respect to CD68 expression, WBI induced somewhat smaller increases in the density and percentage of ED1^+^ cells in those regions and at those ages characterized by higher basal expression. Whether this reflects senescence that leaves microglia less capable of limiting radiation-induced damage and contributing to repair will require additional studies targeting phenotypic markers that are linked more directly to the trophic and other “positive” functions of microglia.

### Oligodendrocyte precursor cells

White matter changes are not always seen in irradiated patients or animals that show cognitive deficits [25]–[27], but this may be because clinical imaging and typical histological evaluation have limited ability to assess oligodendrocyte turnover and some changes in structure that may affect function [71]. A variety of studies indicate that changes in oligodendrocytes contribute to radiation-induced neural deficits and cognitive dysfunction [32], [33]. Thus, any aging-related changes in the oligodendrocyte population likely affect vulnerability to normal tissue damage. Regulation of oligodendrocyte turnover may be particularly critical. The combination of immunomarkers used in this study permitted analysis of a population of proliferating cells that appear to be primarily OPCs. Consistent with evidence that most cell proliferation in the normal adult brain involves cells in the oligodendrocyte lineage [28], [30], [72], we observed that Ki67^+^ cells that did not express Iba1 were virtually always labeled by the NG2 antibody, a common marker of OPCs [24], [73]. It now is recognized that NG2^+^ cells have greater lineage plasticity than previously appreciated [74], [75]. Nevertheless, we showed previously that most dividing cells in white- and gray matter of normal, young adult animals express both NG2 and other markers of OPCs [31]. We also demonstrated here that NG2^+^ cells (in young adult and old, control and irradiated rats) that were not Iba1^+^ expressed the OPC marker PDGFR-a. Thus, the majority of Ki67^+^/Iba1^−^ cells we analyzed were proliferating OPCs (although some may have the potential to produce other cell types, see below).

#### Response of OPCs to aging and to WBI

We detected no effect of normal aging on the density of Ki67^+^/Iba1^−^ cells. Thus, the small populations of proliferating OPCs in the hippocampus and much larger population in the CC are stable in size across the rat's adult lifespan.

Proliferating OPCs are transiently but robustly responsive to WBI. The observed increases in proliferation of OPCs in gray- and white matter of the adult rat brain are consistent with previously reported effects in the adult rat spinal cord [73] and six-week-old mouse brain [76]. The proliferation response likely is due, at least in part, to radiation-induced death of oligodendrocytes and/or OPCs. It remains to be demonstrated directly that the proliferative response results in new, mature oligodendrocytes and contributes to myelin repair and maintenance, but that conclusion is consistent with recent evidence that the size of major forebrain commissures and the number of oligodendrocytes is unchanged at 12 months following fractionated WBI in adult rats [27].

Like the responses of microglia, the response of proliferating OPCs to WBI is region- and age-dependent. The relative increase in proliferation one week after WBI is smaller in the CC than in the hippocampus. A high level of basal proliferation associated with normal myelin turnover in white matter may mean that only a modest increase is required to respond to additional damage caused by a single, relatively low dose of irradiation, or other factors may set a limit on the extent of proliferation within the microglial population. Among the regions examined, effects of WBI on proliferating OPCs are greatest in the CA3 region of middle-aged and older rats. Thus, CA3 appears to be differentially sensitive to WBI as well as to aging. The significance of this aging-related increase in WBI-induced proliferation of OPCs in CA3 remains to be established, but it is known that NG2^+^ cells in gray matter interact synaptically with neurons [77] and are capable of producing astrocytes as well as oligodendrocytes, [78]. Thus, the cells may respond to irradiation in ways that are independent of a role as OPCs.

### Summary

Microglia and OPCs play critical roles in the response of the nervous system to irradiation. The dynamic regulation of these cell types differs among neural regions and across the adult lifespan, likely influencing both their vulnerability to radiation-induced changes and their contributions to radiation-induced normal tissue injury. Such regional and-age dependent responses are important considerations in ongoing attempts to understand the neurobiological mechanisms underlying radiation-induced cognitive dysfunction in cancer patients.

## Supporting Information

Figure S1 **Image processing for analysis of Iba1 labeling.** Images from CA1 region of representative sham irradiated (A) and irradiated (B) young adult rats (1 week after WBI). Top panels show z projections of five image planes representing 10 µm section depth. Subsequent panels represent images corrected for background illumination, segmented to separate areas with labeled cells and process from areas without (white in A, dark gray in B), and thresholded to permit measurement of unoccupied areas (black). The percentage of the ROI without label is indicated in the lower right corner of the binary images.
(TIF)

Figure S2 **Area lacking microglial cell bodies and processes.** A. Mean open area in normally aging rats in the young adult (open bars), middle-aged (light gray bars) and old (dark gray bars) groups (values combined for sham irradiated, control rats of each age from the 1- and 10-week survival groups). Mean values (+sem) are indicated. Significant effects of age within each region are indicated within the group of bars representing that region; significant differences among regions at each age are indicated above the bars.B. WBI-induced changes in microglial density at 1 week after WBI. Mean values (+sem) are indicated; open bars indicate no significant difference from age-matched, sham irradiated, controls. Asterisks indicate that the mean for irradiated animals was significantly different from that for age-matched, sham irradiated controls. At 10 weeks post-WBI, there were no significant differences between sham irradiated and irradiated rats in any region at any age (data not shown).
(TIF)

Text S1 **Supplemental materials and methods.**
(DOCX)
