# Supplementary figures and images for: Regionally Distinct Responses of Microglia and Glial Progenitor Cells to Whole Brain Irradiation in Adult and Aging Rats
Source: PLoS One. 2012 Dec 26;7(12):e52728. doi: 10.1371/journal.pone.0052728 (PMC3530502; doi:10.1371/journal.pone.0052728)

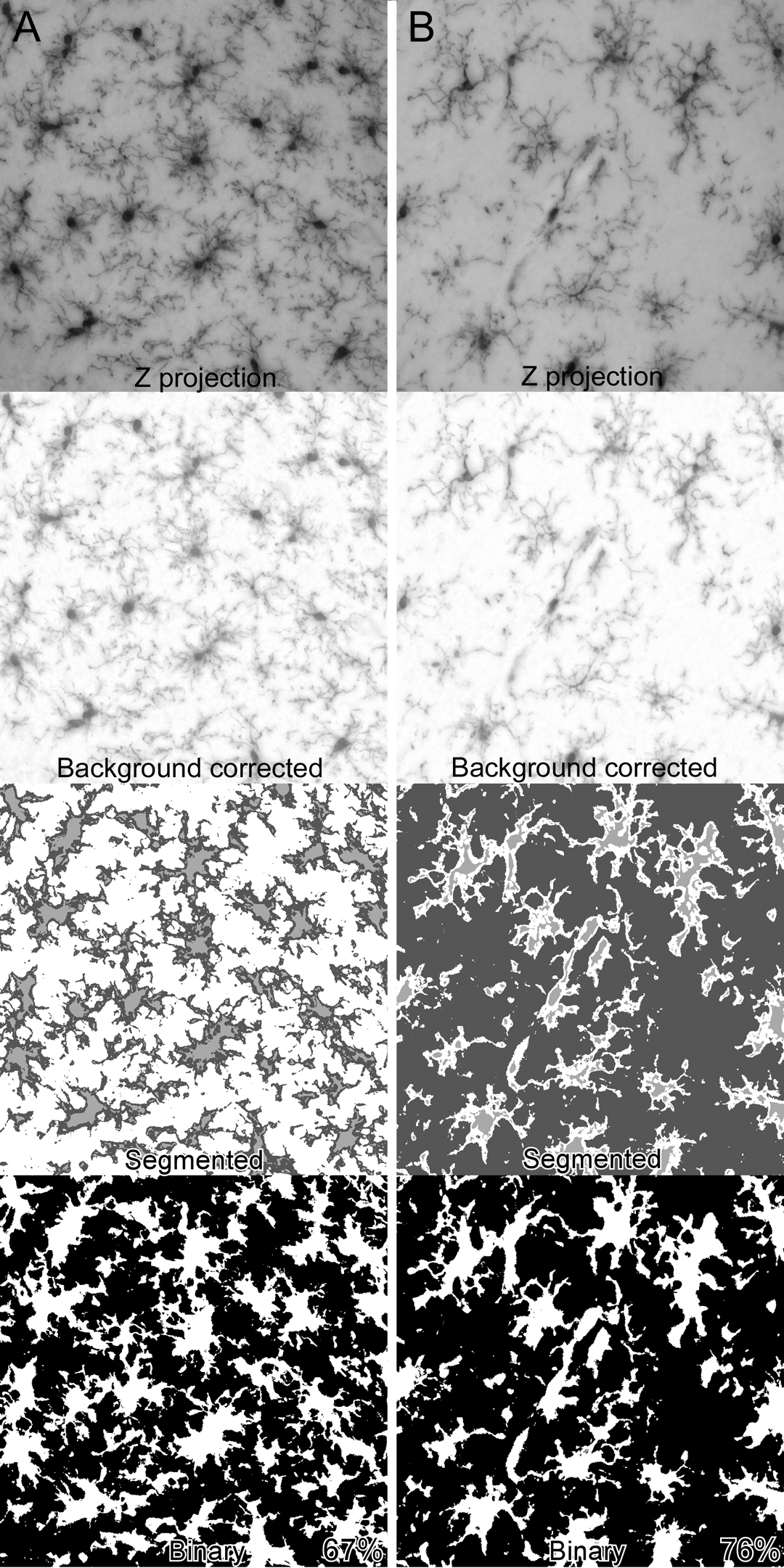

Supplement: Figure S1 — Image processing for analysis of Iba1 labeling. Images from CA1 region of representative sham irradiated (A) and irradiated (B) young adult rats (1 week after WBI). Top panels show z projections of five image planes representing 10 µm section depth. Subsequent panels represent images corrected for background illumination, segmented to separate areas with labeled cells and process from areas without (white in A, dark gray in B), and thresholded to permit measurement of unoccupied areas (black). The percentage of the ROI without label is indicated in the lower right corner of the binary images. (TIF) [file pone.0052728.s001.tif]

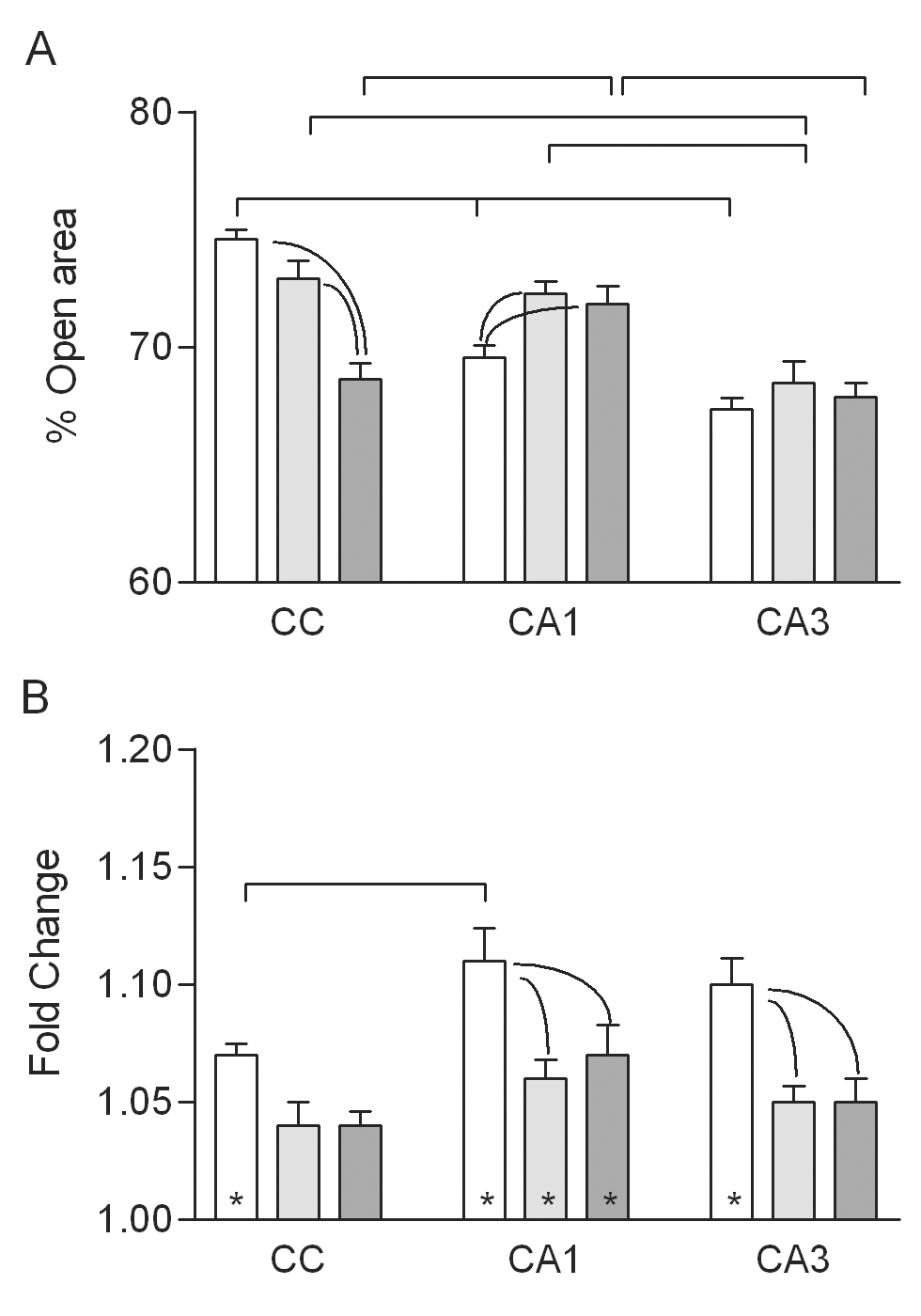

Supplement: Figure S2 — Area lacking microglial cell bodies and processes. A. Mean open area in normally aging rats in the young adult (open bars), middle-aged (light gray bars) and old (dark gray bars) groups (values combined for sham irradiated, control rats of each age from the 1- and 10-week survival groups). Mean values (+sem) are indicated. Significant effects of age within each region are indicated within the group of bars representing that region; significant differences among regions at each age are indicated above the bars.B. WBI-induced changes in microglial density at 1 week after WBI. Mean values (+sem) are indicated; open bars indicate no significant difference from age-matched, sham irradiated, controls. Asterisks indicate that the mean for irradiated animals was significantly different from that for age-matched, sham irradiated controls. At 10 weeks post-WBI, there were no significant differences between sham irradiated and irradiated rats in any region at any age (data not shown). (TIF) [file pone.0052728.s002.tif]
