# Supplementary material for: Regionally Distinct Responses of Microglia and Glial Progenitor Cells to Whole Brain Irradiation in Adult and Aging Rats
Source: PLoS One. 2012 Dec 26;7(12):e52728. doi: 10.1371/journal.pone.0052728 (PMC3530502; doi:10.1371/journal.pone.0052728)
Supplement: Text S1 — Supplemental materials and methods. (DOCX) [file pone.0052728.s003.docx]

**SUPPLEMENTAL MATERIALS AND METHODS**

Details of experimental procedures that have not been reported previously [*1*] are provided below.

*Animals, irradiation procedures, and tissue processing*

Eighty male Fischer 344 × Brown Norway (F×BN) F_1_ hybrid rats were obtained at 7, 17 and 27 months of age and acclimated for 1 month prior to entry into the study. The animal facility at WFUSM is fully accredited by the American Association for Accreditation of Laboratory Animal Care and complies with all Public Health Service-National Institutes of Health and institutional policies and standards for laboratory animal care; the institutional Animal Care and Use Committee approved all protocols. Rats of each age were divided randomly into irradiated and sham control groups and anesthetized with ketamine/xylazine (80/4 mg/kg body weight). Irradiated rats received a 10 Gy dose using a ^137^Cs irradiator with collimating devices to maximize dose to the whole brain and lead shielding to protect the body and eyes. According to the LQ model and assuming late effects in brain tissue, the single 10 Gy dose would be approximately equivalent to 30 Gy in 2 Gy fractions [2]. Rodents are less sensitive than humans to ionizing radiation, however, and it has been argued that, due to differences in radiosensitivity, a 10 Gy dose in rats approximates the biological effects of a single 2-3 Gy fraction in clinical radiotherapy [*3*]. Regardless of the exact equivalency, the 10 Gy dose in rats is in a range that is clinically relevant. Moreover, 10 Gy has been demonstrated to produce cognitive deficits in mice and rats (see Discussion). Sham animals were anesthetized but not irradiated. Irradiated and sham rats of each age were divided into two survival groups (N = 5-7/group) and euthanized at 1 or 10 weeks post irradiation/anesthesia. Following euthanasia with ketamine/xylazine (80/12 mg/kg body weight), brains were removed and the right hemispheres fixed by immersion (4% paraformaldehyde). Serial 40 µm thick coronal sections were cut, collected in an antifreeze solution, and stored at -20ºC until used for immunolabeling.

*Immunohistochemistry and immunofluorescence*

For analysis of microglial number and cell proliferation and phenotype (see below), 1-in-24 series of sections representing the entire anterior to posterior extent of the hippocampus were selected and labeled by immunohistochemistry (IHC) using published protocols [*1*]. The primary antibodies used included: i) rabbit monoclonal anti-Ki67 (SP6 clone, Abcam, 1:3000, which labels dividing cells in all phases of the cell cycle but not cells in G_0_, ii) mouse monoclonal ED1 (to the rat homologue of CD68, Serotec, 0.83µg/ml), which is associated with activated/phagocytic macrophages/microglia, and iii) rabbit polyclonal anti-Iba1 (WAKO, 0.083µg/ml), which labels all macrophages/microglia. The ED1 and Iba1 antibodies do not differentiate between intrinsic microglia and peripheral macrophages, but under the conditions examined here peripherally-derived macrophages likely were rare (see Discussion) so ED1^+^ and Iba1^+^ cells are referred to as microglia. One series of sections was labeled for ED1 alone, using the avidin-biotin peroxidase technique (ABC Elite kit, Vector Laboratories) and diamino-benzidine (DAB, Vector). A second series of sections was double-labeled by sequential immunohistochemistry, first for Ki-67 visualized using Vector SG chromagen (Vector Laboratories), and then for Iba1 visualized with DAB.

For additional assessment of the phenotype of Ki67^+^ cells, tissue sections were processed for triple-immunofluorescent (IF) labeling [*4*] with rabbit monoclonal anti-Ki67 (clone SP6, Novus, 1:200), goat polyclonal anti-Iba1 (Lifespan Biosciences, 0.5 µg/ml), and mouse monoclonal anti-chondroitin sulfate proteoglycan (NG2; clone 132.39, Millipore, 1 µg/ml), commonly used as a marker of oligodendrocyte precursor cells (OPC) [*5*]. Additional sections were processed for IF labeling with mouse monoclonal anti-NG2, goat anti-Iba1, and rabbit anti- rabbit anti-platelet-derived growth factor receptor-alpha (PDGFR-a; Santa Cruz (C-20); 0.2 lg/mL), another marker of OPCs [*6*]. In addition, the relationship between CD-68 expression and microglial morphology was assessed using double-IF labeling with anti-Iba1 and the ED1 antibody. Sections labeled by IF were examined using a Leica SP2 laser scanning confocal microscope and 63x oil immersion apochromatic objective. Analysis of coexpression of Ki67 with Iba1 and NG2 or PDGFR-a and assessment of cell morphology and ED1 labeling were completed without enhancement of the confocal images (levels of some signals were adjusted for presentation, as noted in figure legends).

*Quantitative analyses*

All quantitative analyses were completed blindly using coded sections. For cell counts, every 24^th^ section in systematically random series through the anterior-posterior extent of the dentate gyrus was analyzed. The methods of quantification differed among endpoints depending upon the distribution of cells labeled by each marker. Iba1^+^ cells occurred at high density, were distributed relatively homogeneously within each region of interest (ROI), and were counted using the optical fractionator workflow on the Stereo Investigator system (MBF Bioscience, Williston, VT). The average coefficient of error (Schmitz-Hof) was <0.05 for both hippocampus and CC. Cells labeled by ED1 or Ki67 or double-labeled for Iba1 and Ki67 occurred at low density, were heterogeneously distributed, and were counted using a modification of the optical disector [*7, 8*] and the Neurolucida system for quantitative morphometry (MBF Bioscience) as previously described [4]. Counts are expressed as # of cells/mm^3^ (Iba1^+^, ED1^+^, Ki67^+^, Ki67^+^/Iba1^+^). The percentage of Iba1^+^ cells expressing ED1 and the percentage of Iba1^+^ cells that was proliferating (Ki67^+^) was estimated for each ROI in each rat by dividing the densities of ED1^+^ cells and of Iba1^+^/Ki67^+^ double labeled cells, respectively, by the density of Iba1^+^ cells.

*Image analysis of ED1 labeling*

Brightfield images of Iba1-labeled sections were obtained with an Olympus BX-51 Microscope, 40x/0.75 N.A. objective, and Optronics Microfire digital camera. For each ROI (CC and stratum radiatum of CA1 and CA3) z stacks of 5 images representing 10 μm section depth were collected from each of three sections from each rat (sections representing comparable positions along the anterio-posterior axis of the dorsal hippocampus). Subsequent image processing and analysis were completed using ImageJ [*9*] (Supporting Fig. 1). The five images in each stack were projected to a single plane using a minimum intensity function and corrected for background illumination [*10*]. Note that this approach provides a conservative estimation of the area without microglial processes since the maximum area occupied by labeled cells and processes in each image plane is represented in the projected image. Iba1 labeling then was separated from background in the projected image using the k-means clustering segmentation plugin (three clusters) followed by threshholding to produce a binary image with areas of Iba1 labeling represented by white and areas without labeled cells or processes represented by black. The “measure” function then was used to determine the percentage of the area that was occupied by Iba1^+^ cells and processes. The mean of three measures from each ROI in each rat were analyzed as described in the manuscript (Statistical Analysis) the results are shown in Supporting Figure 2.

**Supporting References**

1. Schindler MK, Forbes ME, Robbins ME, Riddle DR (2008) [Aging-dependent changes in the radiation response of the adult rat brain.](http://www.ncbi.nlm.nih.gov/pubmed/18164853) Int J Radiat Oncol Biol Phys 70: 826-834.

2.Fowler JF (1989) The linear quadratic formula and progress in fractionated radiotherapy. Br J Radiol 62: 679-675.

3. Monje ML, Palmer T (2003) Radiation injury and neurogenesis. Curr Opin Neurol 16(2): 129-134.

4. Lichtenwalner RJ, Forbes ME, Bennett SA Lynch CD, Sonntag WE, et al. (2001) Intracerebroventricular infusion of insulin-like growth factor-I ameliorates the age-related decline in hippocampal neurogenesis. Neuroscience 107: 603-613.

5. Polito A, Reynolds R (2005) [NG2-expressing cells as oligodendrocyte progenitors in the normal and demyelinated adult central nervous system.](http://www.ncbi.nlm.nih.gov/pubmed/16367798) J Anat 207: 707-716.

6. [Hart IK](http://www.ncbi.nlm.nih.gov/pubmed?term=%22Hart%20IK%22%5BAuthor%5D), [Richardson WD](http://www.ncbi.nlm.nih.gov/pubmed?term=%22Richardson%20WD%22%5BAuthor%5D), [Heldin CH](http://www.ncbi.nlm.nih.gov/pubmed?term=%22Heldin%20CH%22%5BAuthor%5D), [Westermark B](http://www.ncbi.nlm.nih.gov/pubmed?term=%22Westermark%20B%22%5BAuthor%5D), [Raff MC](http://www.ncbi.nlm.nih.gov/pubmed?term=%22Raff%20MC%22%5BAuthor%5D) (1989) PDGF receptors on cells of the oligodendrocyte-type-2 astrocyte (O-2A) cell lineage. Development 105(3): 595-603.

7. Gundersen HJ, Bagger P, Bendtsen TF, Evans SM, Korbo L, et al. (1988) [The new stereological tools: disector, fractionator, nucleator and point sampled intercepts and their use in pathological research and diagnosis.](http://www.ncbi.nlm.nih.gov/pubmed/3056461) APMIS 96: 857-881.

8. Kempermann G, Kuhn HG, Gage FH (1998) Experience-induced neurogenesis in the senescent dentate gyrus. J Neurosci 18: 3206-3212.

9. Rasband, W.S., ImageJ, U. S. National Institutes of Health, Bethesda, Maryland, USA, <http://rsb.info.nih.gov/ij/>, 1997-2008.

10. Landini G (2006-2010) How to correct background illumination in brightfield microscopy. Available at: <http://imagejdocu.tudor.lu/doku.php?id=howto:working:how_to_correct_background_illumination_in_brightfield_microscopy>
